# Optimizing the thermophysical behavior of a novel ternary hybrid nanofluid for energy applications through experimental research

**DOI:** 10.1016/j.heliyon.2024.e32728

**Published:** 2024-06-08

**Authors:** Humphrey Adun, Muhammad Abid, Doga Kavaz, Yihua Hu, Juliana Hj Zaini

**Affiliations:** a Operational Research Center in Healthcare, Near East University, TRNC Mersin 10, Nicosia, 99138, Turkey; b Department of Energy Systems Engineering, Faculty of Integrated Technologies, Universiti Brunei Darussalam, Bandar Seri Begawan, Brunei Darussalam; c Bio Engineering Department, Cyprus International University, Haspolat-Lefkosa, Mersin 10, KKTC, Turkey; d Electrical Engineering Department, King's College London, United Kingdom

**Keywords:** Ternary nanofluid, Density, Enhancement, Particle size, Volume fraction, Nanofluid

## Abstract

The continual use of fossil fuel technologies has negatively impacted on the environment and has caused huge health challenges globally. Despite the growth of renewable energy technologies, their efficiency issues have hindered widespread adoption. The use of nanofluids as heat transfer fluids in renewable energy technologies have further improved their overall efficiency, resulting in a more environmentally friendly performance of these systems. For automotive fuel and coolant systems, hybrid nanofluids are gaining appeal due to their remarkable ability to enhance thermal performance and accelerate heat transfer rates. Ternary-hybrid nanofluids, which combines three different types of nanoparticles in a wide range of mixing ratios, are an intriguing but mostly speculative concept. Optimizing the mixing ratio for effective heat transfer characteristics is important for energy applications. A unique Al_2_O_3_/ZnO/Fe_3_O_4_ ternary nanofluid is synthesized and its density is measured in this investigation. The nanofluid preparation included three different mixing ratios (1:1:1, 2:1:1, and 1:2:1), with the volume fraction between 0.5 % and 1.25 %. This study also includes a discussion of the density prediction analysis. The result shows that at a temperature of 25 °C and a volume fraction of 1.25 %, the maximum density is determined to be 1165 kg/m^3^. The Random Forest algorithm gives the best prediction accuracy with an R^2^ value of 0.928.

## 1 Introduction

The utilization of fossil fuels has continuously increased over the years, despite the development of renewable energy technologies [1]. This is due to several reasons: the rising global energy demands, the relatively low cost of fossil fuels, and the established infrastructure that supports their extraction, distribution, and consumption. Additionally, the slow pace of innovation and adoption of renewable energy technologies has made it difficult for them to compete with the efficiency and reliability of fossil fuels. This ongoing reliance on fossil fuels has significant environmental and health implications, contributing to global warming, air pollution, and related health issues. Furthermore, technological advancements such as blockchain technology necessitate large computing installations, thereby increasing the need for cooling and consequently elevating electricity demand. . As international organizations advocate for more sustainable energy production, the focus on efficient heat transfer fluids (HTFs) becomes essential, given their critical role in large-scale renewable energy systems. The advancement of solar thermal technologies and photovoltaic (PV) systems, which have the potential to gradually replace conventional power plants, is contingent on the efficiency of heat transfer fluids [2]. This is because HTFs enhance the efficiency of these technologies, thereby reducing the cost-to-generation metric compared to conventional systems.

The thermophysical properties of fluids are crucial for the development of high-efficiency heat transfer technologies. Although frequently used, HTFs such as water, motor oil, glycerol, and ethylene glycol (EG) each possess their own set of advantages and disadvantages [3]. As technology rapidly advances, more heat is generated in innovative heat transfer systems, such as microelectromechanical devices and high-efficiency heat exchangers, leading to an increasing need for effective heat transfer fluids (HTFs) [4]. Conventional fluids typically exhibit poor thermal conductivity compared to solids, a critical factor in determining the efficiency of heat transfer fluids (HTFs) [5]. Several studies have utilized nanofluids in the engineering field. A study by Gokhan et al. [6] gave a comprehensive review of the nanofluid application in the refrigeration system.

The addition of solid particles has been shown in experimental works to improve the heat transfer characteristics of conventional fluids. Dispersion of micrometre or millimetre-sized solid particles in fluids has its theoretical background in Maxwell's work from 1873 [7]. Numerous theoretical and practical investigations have explored the potential of particles at the millimetre or micrometre scale suspended in fluids to enhance their thermal conductivity. However, there are limitations concerning clogging, wearing, considerable pressure reduction, stability, and sedimentation caused by these solid particles [8]. To address these challenges and enhance thermal conductivity in heat transfer fluids, Choi [9] conducted experiments with the use of nanometer-sized particles, commonly referred to as nanoparticles. Numerous researchers have experimented with nanoparticles suspended in various base fluids [10]. The properties of nanofluids have been discovered to be affected by a wide range of variables, including nanoparticle volume fraction, temperature, nanoparticle size, nanolayer thickness, base fluid thermal conductivity, nanofluid pH, and nanoparticle thermal conductivity [11]. A study by Bhattad & Sarkar [12] investigated the effect of particle size and shape on the performance of refrigeration systems. Their study showed that the thermal performance increased with particle size, and the particle shape affected the performance due to the surface area-to-volume ratio.

Since nanofluids are constrained by the nanoparticles' chemical characteristics, researchers have turned to hybrid nanofluids for further experimentation [13]. Non-metallic oxides exhibit superior thermal conductivity but have lower chemical stability, whereas metallic oxides, despite their poor thermal conductivity, show excellent chemical stability. Moreover, the synthesis of hybrid nanocomposites can present several challenges. For instance, a composite of oxide and ferrous nanoparticles often results in a high tendency for agglomeration, which can be mitigated by employing surfactants. Additionally, ferrous materials may cause corrosion, especially when mixed with oxides; overcoming this issue may involve the use of corrosion inhibitors.

The heat transfer characteristics and practical uses of nanofluids have been enhanced by hybridization. Thermophysical property enhancements of hybrid nanofluids have been the subject of several reviews [14,15]. Recently, scientists have turned their attention to studying ternary nanofluids, which are mixtures containing three different nanoparticles suspended in a base fluid. Cakmak et al. [16] conducted experiments using sol-gel-prepared ternary nanocomposites of rGO-Fe_3_O_4_–TiO_2_/ethylene glycol. FTIR, SEM, EDX, XRD, and Zeta potential were used to determine the morphology of the rGO-Fe_3_O_4_–TiO_2_ nanocomposites. The thermal conductivity was tested across a range from 25 to 60 °C. The findings of their research highlighted a significant correlation between the volume fraction and the temperature-related increase in thermal conductivity of rGO-Fe_3_O_4_–TiO_2_/ethylene glycol (EG) nanofluids. Notably, there was a 13.3 % enhancement in thermal conductivity at 60 °C when the nanofluid had a concentration of 0.25 wt per cent. They also discovered that the ternary hybrid nanofluid exhibited sufficient stability for use in both heating and cooling applications. In the research conducted by Sahoo et al. [17], the viscosity of Al_2_O_3_/CuO/TiO_2_ ternary nanofluid was studied, with a focus on assessing the effects of temperature and volume fraction. Their experiments were carried out at temperatures ranging from 35 to 50 °C with volume fractions between 0.01 and 0.1 %. The findings indicated that an increase in the solid particle percentage within the nanofluid led to a rise in its dynamic viscosity. Additionally, the experiments demonstrated that the maximum increase in dynamic viscosity occurred at a 0.1 % volume fraction, showing a 55.41 % enhancement compared to a water-based Al_2_O_3_–TiO_2_ hybrid nanofluid and a 17.25 % enhancement compared to a water-based Al_2_O_3_–CuO hybrid nanofluid at 45 °C. In the study conducted by Dezfulizadeh et al. [18], experiments were carried out to investigate the dynamic viscosity and thermal conductivity (k_nf) of a ternary hybrid nanofluid composed of Cu–SiO_2_-MWCNTs suspended in water. The tests were conducted at temperatures ranging from 15 to 65 °C and with volume fractions between 1 % and 3 %. It was observed that both the dynamic viscosity and thermal conductivity of the ternary hybrid nanofluid varied with concentration and temperature. Compared to mono and binary nanoparticles, the ternary hybrid nanofluid exhibited significantly higher thermal conductivity and dynamic viscosity. In the research conducted by Ahmed et al. [19], an experimental investigation focused on the turbulent heat transfer capabilities of Al_2_O_3_@TiO2–ZnO/DW (distilled water) nanofluid. In their experiment, the nanofluid was exposed to continuous heat flow conditions within a closed circular heat exchanger. Absorption and uniform dispersion of the nanofluid were confirmed through UV–Vis and FESEM analyses, using the two-step preparation method. Ahmed et al. observed that an increase in the Reynolds number from 5849 to 24,544 led to enhanced heat transfer coefficients and Nusselt numbers for the Al_2_O_3_@TiO_2_–ZnO/DW-based ternary composite nanofluids. These improvements were noted across various weight percentages—0.1, 0.075, 0.05, and 0.025—without the need for any surfactant application. At 45 °C, the nanofluid with a 0.1 wt per cent showed an increase in effective thermal conductivity recorded up to 1.14 W/m.K, an increase of 69 % compared to the base fluid. This was measured using constant Reynolds numbers and heat flux. Compared to the base fluid, the nanofluid showed a 79 % improvement in heat transfer efficiency at a volume fraction of 0.1. The researchers concluded that the composite of nanoparticles of varying shapes and sizes was responsible for the improvement. There is also evidence from their study to show that this novel ternary composite nanofluid based on metal oxides showed better thermophysical and heat transfer capabilities, hence, making it a promising candidate for more efficient energy transmission. The study by Mirsaeidi et al. [20] conducted a review of the thermophysical properties of mono and hybrid nanofluids, placing particular emphasis on density properties. Their review noted that, in contrast to thermal conductivity, the density of nanofluids decreases with an increase in temperature.

Despite the extensive research on the thermophysical properties of nanofluids, including thermal conductivity [[21], [20], [22]], viscosity [23,24], and specific heat capacity [25,26], one of the most significant physical characteristics, density, has not yet been explored in detail. In most cases, the density of nanofluid is determined using the classical mixture density model (linear approach for densities and volume fractions) for the conventional solid-liquid mixture [27]. This model does not account for the nanolayer formed by the nanoparticles, which may affect the density of the fluid. The enhanced performance of nanofluids in heat transfer has been the focus of a great deal of study in recent years, and their superior thermal conductivity [28] over traditional fluids has been shown via several experiments. However, there is still a need for further research into the characteristics of nanofluids to fully understand these materials and their potential industrial uses [27]. As solids have a greater density than liquids, the nanofluid mixture's density may be enhanced by adding a few solid nanoparticles to the base liquid [29]. A liquid density meter was used in research by Ref. [30] to determine the density of alumina (Al_2_O_3_) (DA-130 N, KEM, Japan). According to their findings, a density of 1.26 kg/m^3^ was reached for a weight percentage of 1.5 wt%. Furthermore, across all experimented volume concentrations, it was observed that density decreases as temperature increases.

As researchers continue to synthesize hybrid and ternary nanofluids, there arises a pressing need for further discussion on the density of nanofluids. The dissemination of findings related to the density of nanofluids will be crucial for the development of machine-learning models aimed at predicting the density of nanofluids. Therefore, our research fills a notable gap in the existing body of knowledge by synthesizing a novel ternary nanocomposite and experimentally estimating its density. The mixing ratio is also taken into account since it is a relatively unexplored aspect of nanofluid synthesis. In this research, we developed density-predictive algorithms for the Al_2_O_3_–ZnO–Fe_3_O_4_ ternary nanofluid. This is instrumental for forecasting the behaviour of nanofluids in future applications by comparing their predicted results with those previously published. Table 1 shows the experimental synthesis of hybrid nanofluids and their density measurements.

**Table 1:** Experimental analysis of the density of hybrid nanofluid.

References	Nanoparticles	Mixing ratio	Base fluid	Volume fraction	Temperature range	Density measurement	Correlation equation
[31]	Graphene, Silver	50:50	Water	0.02 %, 0.04 %, 0.06 %, 0.08 %, 0.1 %	20 °C, 25 °C, 35 °C, 40 °C.	Mettler Toledo DE-40 density meter	–
[32]	MWCNT, Fe_3_O_4_	50:50	Distilled water	0.05 %–0.3 %	20–60 °C	Mixture laws	$\rho_{nf}={\left(1-\emptyset \right)}_{\rho_{water}}+{\rho \emptyset }_{water}+{Fe}_{3}O_{4}$
[33]	Al_2_O_3_/MWCNT	80:20	Water	0.25 %–2 %	25 °C–50 °C	SVM-3000, Anton Paar	–
[33]	ZnO/MWCNT	80:20	Water	0.25 %–2 %	25 °C–50 °C	SVM-3000, Anton Paar	–
[33]	TiO_2_/MWCNT	80:20	Water	0.25 %–2 %	25 °C–50 °C	SVM-3000, Anton Paar	–
[33]	Ce_2_O_3_/MWCNT	80:20	Water	0.25 %–2 %	25 °C–5 0°C	SVM-3000, Anton Paar	–
[34]	TiO_2_–SiO_2_	20:80	Water/Green Bio-Glycol	0.5 %–3 %	30 °C–70 °C	Portable Density meter (Model: DA-130 N)	
[35]	rGO/CO_3_O_4_	50:50	Water	0.05 %, 0.1 %, and 0.2 % wt%	20, 30, 40, 50, and 60 °C	Differ-ential scanning calorimeter (DSC 2920 modulated, TA Instruments, New Castle, DE)	
[36]	MgO/TiO_2_	50:50, 80:20, 20:80, 60:40 and 40:60	Distilled water	0.1 %–0.5 %	15 °C–60 °C	density meter (DMA 4500 M, Anton Paar Company, Austria)	
[37]	Al_2_O_3_/SiO_2_	50:50	Water	0.05 %, 0.1 % and 0.2 %)	20 °C–60 °C	Anton Paar density meter (DMA 45)	
[38]	CNT/Al_2_O_3_	50:50	Water	0.05 and 0.1 %	300 K–350 K		
[39]	Al_2_O_3_/SiC	50:50	Water/EG (60:40, 50:50, 60:40, 50:50)	0.4 and 0.8 %	20 °C–75 °C	density meter DMA 35 (Anton Paar/China)	
[19]	ZnO/Al_2_O_3_/TiO_2_	Equal mixture	Water	0.025, 0.05, 0.075, and 0.1 %	20–45 °C		
[40]	CuO/MgO/TiO_2_ ternary	A (33.4 mass% CuO/33.3 mass% MgO/33.3 mass% TiO_2_), B (50 mass% CuO/25 mass% MgO/25 mass% TiO_2_), C (60 mass% CuO/30 mass% MgO/10 mass% TiO_2_), D (25 mass% CuO/50 mass% MgO/25 mass% TiO_2_) and E (25 mass% CuO/25 mass% MgO/50 mass% TiO_2_)	Water	0.1–0.5 %	15–60 °C	Anton Paar digital density meter (DMA 4500 M, Anton Paar Company, Austria)	

The novelty of this study lies in the synthesis of a novel ternary nanocomposite and its experimental density analysis. Given the significance of nanofluids' density in their application as heat transfer fluids, this research is particularly important as it provides insights into the effects of incorporating more than one type of nanoparticle in the synthesis process, a common practice in the literature yet lacking detailed exploration regarding density. The discussion on the density of ternary nanofluids is notably absent in the existing literature, making this work a valuable contribution to the future development of nanofluids and their applications as heat transfer fluids. Furthermore, to the best of the authors' knowledge, no prior studies have focused on the synthesis of an all-metallic oxides nanocomposite mixture and its density evaluation, marking a significant advancement in the field.

## 2 Experimental analysis and machine learning model of density of Al_2_O_3_/ZnO/Fe_3_O_4_ novel ternary nanofluid

### 2.1 Preparation of Al_2_O_3_ nanoparticles

To prepare the aluminium oxide nanoparticles, 0.1 M of aluminium chloride was dissolved in a mixture of 140 ml of deionized water and 10 ml of liquid ammonia. The solution was then stirred for 2 h using a magnetic stirrer and left to age for an additional 4 h. This resulted in the formation of white-coloured precipitates. The mixture was then centrifuged at 12,000 rpm for 15 min, and the precipitate was isolated, washed three times with ethanol to remove any organic impurities, and finally calcined at 800 °C for 1 h in a muffle furnace.

### 2.2 Preparation of Fe nanoparticles

The preparation of the Fe nanoparticle follows the synthesis method of Okonkwo et al. [41]. Fe nanoparticles were prepared by first collecting Olea europaea leaves from a farm and washing them with distilled water to remove dirt. The leaves were then cut into fine pieces, dried for 24 h in an oven at 40 °C, and crushed using an electric blender. 10 g of the crushed leaves were then immersed in 100 ml of ethanol, extracted using a rotary evaporator, filtered, and stored in a cool, dry place before being characterized and synthesized into nanoparticles.

### 2.3 Preparation of ZnO nanoparticles

The ZnO nanoparticles were synthesized by using a straight precipitation approach, with zinc nitrate tetrahydrate (Zn (NO_3_)_2_.4H_2_O) serving as the precursor material and sodium hydroxide (NaOH) acting as the accelerating agent. At 70 °C, using a magnetic stirrer, 150 ml of 0.2 M Zn (NO_3_)_2_.4H_2_O solution was vigorously agitated in a 250 ml Erlenmeyer flask, and then 0.4 M NaOH solution was added dropwise at 80 °C, causing the solution's colour to change from clear to white hazy. After 2 h of stirring, the precipitate was placed in an ice bath to solidify. The mixture was centrifuged at 5000 rpm for 15 min, and the supernatant was skimmed off. Multiple rinses with double-deionized water were used to purge the precipitate of any remaining contaminants. After 3 h of calcining at 500 °C, the result was crushed into a powder and saved for analysis.

Equation (1) shows the experimental required nanoparticle mixture for synthesizing the ternary nanofluid.

(1) $$\varphi =\frac{\left[\frac{w_{{{\text{Al}}_{2}O}_{3}+{\text{Fe}}_{3}O_{4}+\text{ZnO}}}{\rho_{{\text{Al}}_{2}O_{3}+{\text{Fe}}_{3}O_{4}+\text{ZnO}}}\right]}{\left[\frac{w_{{\text{Al}}_{2}O_{3}+{\text{Fe}}_{3}O_{4}+\text{ZnO}}}{\rho_{{\text{Al}}_{2}O_{3}+{\text{Fe}}_{3}O_{4}+\text{ZnO}}}\right]+\left[\frac{w_{water}}{\rho_{water}}\right]}\;x\;100$$

### 2.4 Preparation of the novel ternary nanofluid

In this experiment, a ternary hybrid nanofluid was synthesized by mixing equal proportions of aluminium oxide (Al_2_O_3_), zinc oxide (ZnO), and iron nanoparticles into 100 ml of water in a beaker using a magnetic stirrer. The mixture was subsequently dispersed using a two-stage ultrasonic device to ensure even distribution of the nanoparticles and prevent clumping. The resulting nanofluid demonstrated stability, with no visible nanoparticle agglomeration observed after one week. To obtain various volume fractions of the ternary nanofluid, specific masses were precisely weighed using a highly accurate digital scale.

In this study, three different mixing ratios of Al_2_O_3_–ZnO–Fe_3_O_4_ were synthesized using the two-step method. The first ratio was 1:1:1, with each component making up 33.3 % of the mixture, while the second ratio was 2:1:1, with Al_2_O_3_ making up 50 %, ZnO 25 %, and Fe_3_O_4_ 25 %. The third mixing ratio is 1:2:1. The nanofluids were then made at temperatures between 25 and 65 °C and volume fractions between 0.5 and 1.25 %. 100 ml of water was combined with equal parts of aluminium oxide, zinc oxide, and iron oxide, and then stirred with an ultrasonic vibrator for 5 h to achieve consistency. The volume fraction of the solid particles was estimated using Equation (1). A zeta sizer revealed that the average size of the ternary nanocomposite is 89 nm. Table 2 displays the results of the Zeta potential analysis, which was performed to determine the nanofluid's stability on days 7 and 14. Nanofluid stability may degrade over time due to agglomeration, therefore monitoring their stability is essential.

**Table 2:** Stability test on the Al_2_O_3_/ZnO/Fe_3_O_4_ nanofluid.

Nanoparticles	Volume fraction	7-day Zeta potential (mV)	14-day Zeta potential (mV)
Al_2_O_3_: ZnO: Fe_2_O_3_	0.005	−32	−31
	0.0075	−30	−30
	0.01	−30	−28
	0.0125	−28	−26

**Fig. 1:**
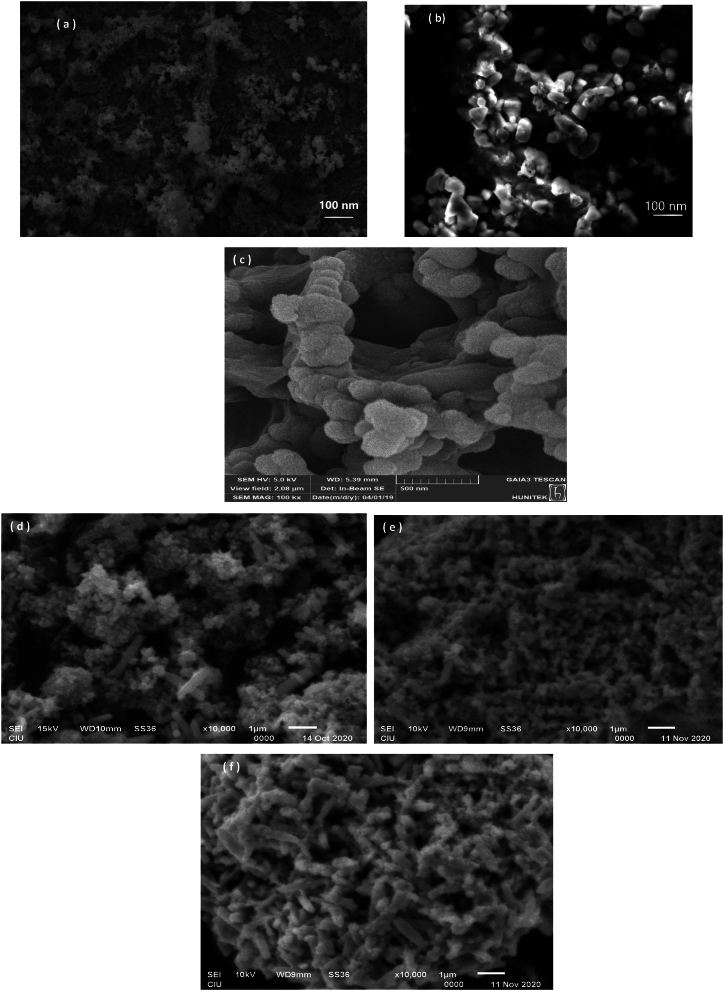
SEM image of (a) Fe_3_O_4_ (b) Al_2_O_3_ (c) ZnO (d) novel ternary Nano-composite (e) Al_2_O_3_–ZnO Nano-composite (f) Fe_3_O_4_–ZnO Nano-composite.

Fig. 1(a–f) illustrates the SEM images of the nanoparticles, the Al_2_O_3_–ZnO–Fe_3_O_4_ nanocomposite, Al_2_O_3_–ZnO nanocomposite, and ZnO–Fe_3_O nanocomposite. The images demonstrate a well-distributed and orderly arrangement of the ZnO–Fe_3_O_4_ and Al_2_O_3_–ZnO nanocomposites. A stacking of the nanoparticles is also seen in the SEM picture of the ternary nanocomposite shown in Fig. 1. Nonetheless, the figures show a significant amount of particle agglomeration, which is indicative of strong van der Waals forces at the particles' surfaces.

**Fig. 2:**
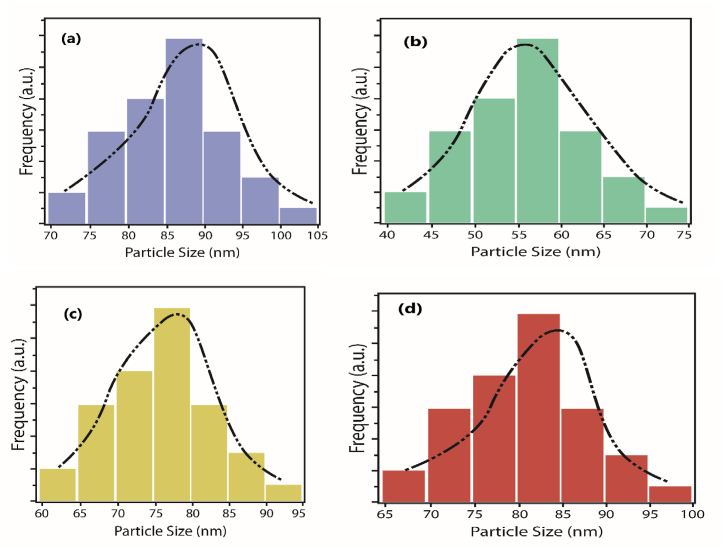
Particle size distribution of (a) novel ternary composite (b) ZnO–Fe_3_O_4_ Nanocomposite (c) Al_2_O_3_–ZnO Nanocomposite (d) Al_2_O_3_–Fe_3_O_4_ Nano-composite.

Fig. 2 displays the results of a Zetasizer nanoparticle analyzer (series Malvern nano ZS). The instrument performs size measurements using a process called ′Dynamic Light Scattering (DLS) also known as PCS – Photon Correlation Spectroscopy, which measures Brownian motion and relates this to the size of the particles. It does this by illuminating the particles with a laser and analyzing the intensity fluctuations in the scattered light. Fig. 2(a and b) shows the distribution of the particle size given in the ZetaSizer. The particle size is retrieved from the peak of the distribution.

The size analysis of the ternary nano-composites is: Al_2_O_3_–ZnO–Fe_3_O_4_, ZnO–Fe_3_O_4_, Al_2_O_3_–ZnO, and Al_2_O_3_–Fe_3_O_4_ were found to have average sizes of 89 nm, 56 nm, 78 nm, and 84 nm, respectively.

## 3 Experimental result of density of novel ternary nanofluid

The density of the ternary nanofluid was determined using a density meter (DMA 4500 M, Anton Paar Company), with a precision of $\pm 5\times 10^{-5}g/{cm}^{3}$. The device's accuracy was verified by using distilled water and comparing the results with established standards [42]. Calibration is essential for precision. The measuring cell of the density meter was cleaned to remove any residues that could affect the accuracy of the measurements. After completing the cleaning process, we activated the device and allowed it to stabilize for at least 30 mins. This stabilization period is essential to ensure accurate performance during the calibration process. The primary focus was on maintaining a controlled environment in terms of temperature and humidity to eliminate any potential external influences. For this calibration, water and air were used as calibration standards due to their well-established and distinct density values. By choosing these two standards, a range of expected measurements was covered. Before the experiment, it was ensured that the water used was highly pure and that both calibration standards were free, from any impurities. The calibration process began by introducing air into the measurement cell of the DMA 4500 M. After a period of adjustment, the meter oscillation period was recorded. Using the known density value of air, the first calibration coefficient was calculated. Next, water was added to the measurement cell and recordings of its oscillation period were made after it had reached equilibrium. Using the known density value of water, the second calibration coefficient was determined. After precisely determining both calibration coefficients, the DMA 4500 M was configured to convert measurements of the oscillation period to accurate density values. To validate the precision of the calibration, a density measurement of a mixture of 60:40 ethylene glycol to water (EG/W) was conducted. Through a comparison between the density measurements obtained from the DMA 4500 M, the values obtained from the ASHRAE were significantly close, with an average error of 1.2 % across the temperature of 10–50 °C considered.

### 3.1 Effect of volume fraction and temperature on the density of novel ternary nanofluid

**Fig. 3:**
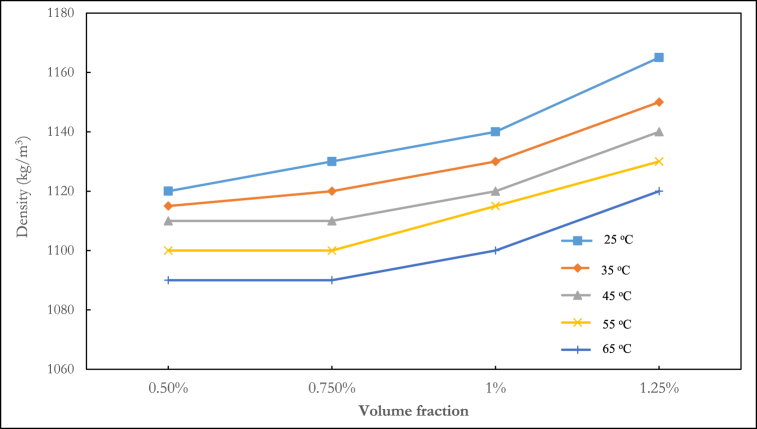
Experimental result of the density of ternary nanofluid (1:1:1).

**Fig. 4:**
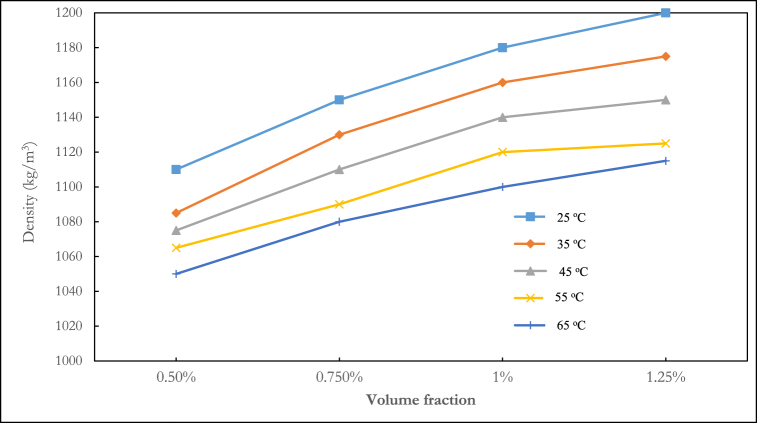
Experimental result of density of ternary nanofluid (2:1:1).

**Fig. 5:**
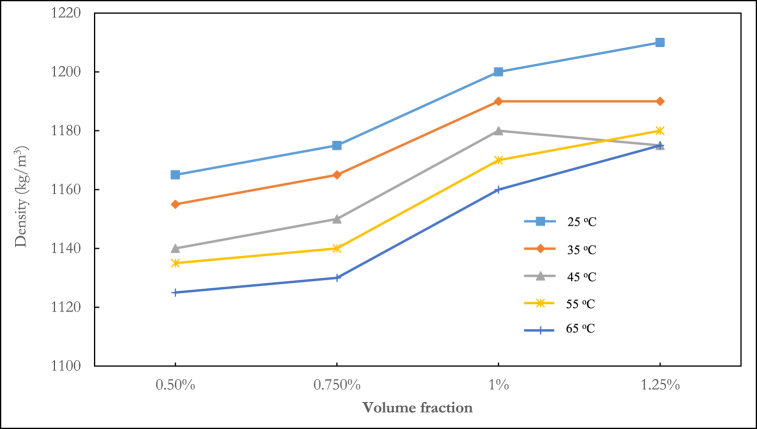
Experimental result of density of ternary nanofluid (1:2:1).

The density of the ternary nanofluid at various volume fractions and temperatures is shown in Fig. 3, Fig. 4, Fig. 5. In Fig. 3, the density fluctuation of the mixing ratio of 1:1:1 Al_2_O_3_–ZnO–Fe_3_O_4_ is displayed. At any temperature, water is shown to have a lower density than nanofluids. The density rises with increasing volume fraction. The highest density recorded was 1165 kg/m^3^ at a temperature of 25 °C and a volume fraction of 1.25 %. At temperatures of 25 °C, 35 °C, 45 °C, 55 °C, and 65 °C, the 1:1:1 mixing ratio at a volume fraction of 1.25 % led to density increases of 14.4 %, 13.6 %, 13.13 %, 12.8 %, and 12.5 % respectively, relative to water. The mixture ratio of 2:1:1 shown in Fig. 4 follows a decreasing density pattern with increasing temperature. This agrees with the density behaviour of hybrid nanofluids reported in the literature.

### 3.2 Effect of mixing ratio on the density of Al_2_O_3_–ZnO–Fe_3_O_4_ ternary nanofluid

**Fig. 6:**
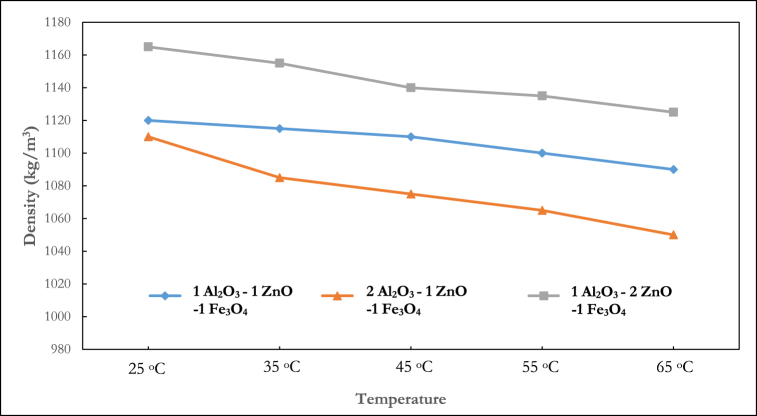
Density of ternary nanofluid at varying mixing ratios (0.5 % volume fraction).

**Fig. 7:**
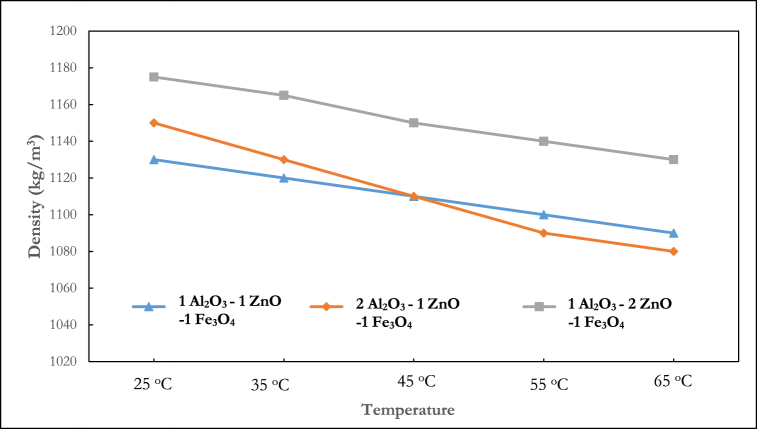
Density of ternary nanofluid at varying mixing ratios (0.75 % volume fraction).

**Fig. 8:**
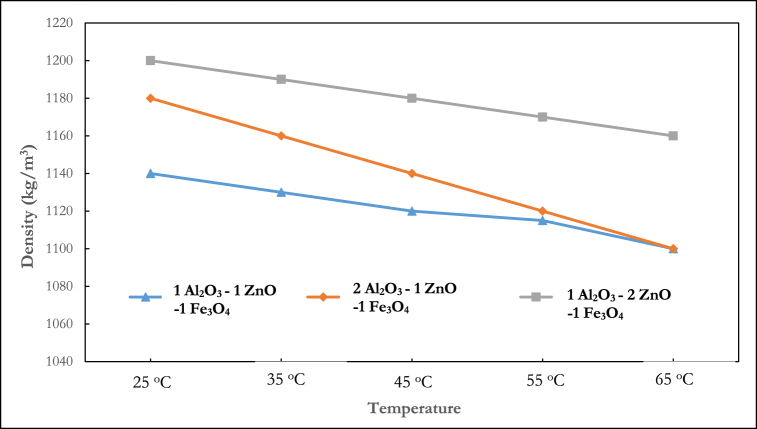
Density of ternary nanofluid at varying mixing ratios (1 % volume fraction).

**Fig. 9:**
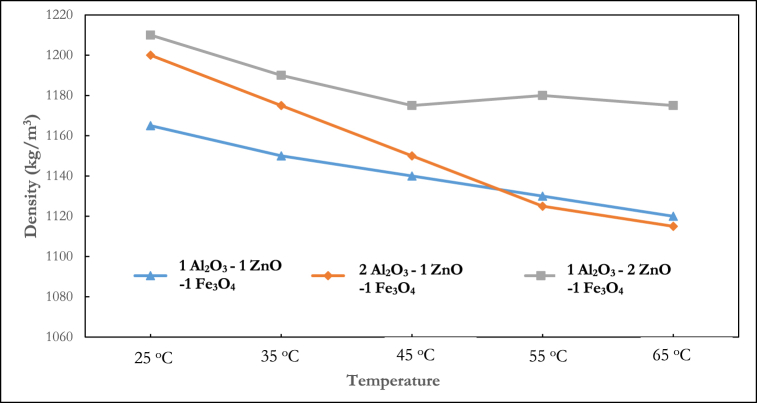
Density of ternary nanofluid at varying mixing ratios (1.25 % volume fraction).

Previous research [43] has demonstrated that the mixing ratio of nanoparticles is a crucial aspect in hybrid nanocomposite manufacturing because it impacts the thermophysical characteristics. Kumar et al. [44] explained that the optimization of the mixing ratio improves the hydrothermal performance of nanofluids. Several studies have considered this phenomenon for thermal conductivity [45], viscosity [46] and specific heat [47] analysis, but no study has experimented with their density. The results shown in Fig. 6, Fig. 7, Fig. 8, Fig. 9 present the density behaviour of Al_2_O_3_–ZnO–Fe_3_O_4_ ternary nanofluid at three mixing ratios of 1:1:1, 2:1:1, and 1:2:1.

In Fig. 6, which shows the density at 0.5 % volume fraction, it is seen that the density of the 1:2:1 Al_2_O_3_–ZnO–Fe_3_O_4_ showed the maximum density across all the temperature ranges. At 25°C, the density measured for the 1:1:1, 2:1:1, and 1:2:1 was 1120 kg/m^3^, 1110 kg/m^3^ and 1165 kg/m^3^ respectively. At 0.75 % volume fraction (Fig. 7), a similar pattern as the 0.5 % volume fraction is observed, with the 1:2:1 mixing ratio having the highest density. However, a deviation is noticed at temperatures of 25 °C and 35 °C, where the 1:1:1 showed a lesser density than the 2:1:1 mixing ratio. For the 0.75 % volume fraction, at 25 °C, the density measured for the 1:1:1, 2:1:1, and 1:2:1 was 1130 kg/m^3^, 1150 kg/m^3^, and 1175 kg/m^3^ respectively.

For the 1 % volume fraction as shown in Fig. 8, the 1:2:1 mixture ratio is observed to have the highest density. At 55 °C, the densities of the 1:1:1, 2:1:1, and 1:2:1 mixing ratio is 1115 kg/m^3^, 1120 kg/m^3^, and 1170 kg/m^3^ respectively.

Fig. 9 shows the density of a 1:2:1 mixing ratio at 25 °C, which is 1210 kg/m^3^. This is the maximum density of any mixture tested at this volume fraction. Except at 55 and 65 °C the lowest density is observed for the 1:1:1 mixing ratio. Density measurements at 25°C show that a nanocomposite with a 1:1:1 mixing ratio has a density of 1165 kg/m^3^, while at a 2:1:1 and 1:2:1 mixing ratio, the densities are 1200 kg/m^3^ and 1210 kg/m^3^ respectively.

It may be concluded that the 1:1:1 exhibits the least density behaviour at greater volume fractions. It is an important conclusion drawn from the research, especially when contrasted to the author's earlier material on heat capacity and viscosity property of Al_2_O_3_–ZnO–Fe_3_O_4_ ternary nanofluid [[46], [47], [48]]. We found in earlier studies that the nanocomposites with a composition of 1:1:1 mixing ratio showed the highest thermal conductivity. Higher volume fractions of ternary nanofluids lead to enhanced heat transfer performance compared to lower concentrations, due to their increased thermal conductivity and reduced density at 1:1:1 mixing ratios.

## 4 Correlation and machine learning prediction of the density of novel ternary nanofluid

Numerically calculating fluid densities has been a subject of study for many years. Research has demonstrated that the density of nanofluids is influenced by several factors, including temperature and volume fraction. As a result, correlation equations that describe the relationship between these variables have been developed. Before the development of more refined methods, density estimates of nanofluids were made using a classical model proposed by Pak and Cho. Subsequent studies have demonstrated significant deviations between the Pak and Cho model's predictions and experimental values. This discrepancy arises because the model overlooks factors such as temperature, particle size, and mixing ratio, all of which play crucial roles in determining the volume and, consequently, the density of nanofluids. We will compare the regression equation, derived from multiple linear regressors based on experimental data, with results obtained from machine learning methods. This comparison aims to evaluate the accuracy and efficiency of traditional statistical models against modern machine-learning approaches in predicting the properties of nanofluids.

In this research, Python-based regression techniques were employed as the machine learning models, selected for their ability to capture both linear and non-linear relationships between input and output variables. The algorithms utilized include Support Vector Regressors, Random Forest algorithms, Ridge algorithms, and Multiple Linear Regressors. These methods were chosen for their robustness and versatility in handling the complex dynamics of nanofluid properties. The mathematical formulations of these algorithms can be retrieved from Refs. [[49], [50], [51]].

### 4.1 Data utilized

There are a total of 60 data considered in the predictive analysis. The inputs are the operating temperature, volume fraction, and mixing ratio retrieved in this study. In contrast to other studies, this incorporates a mixing ratio into the prediction model, which represents the new ground. Seventy per cent of the data is used for training purposes, while the remaining thirty per cent is used for tests. Root-mean-squared error, mean-absolute error, and correlation are used to evaluate the accuracy of a prediction. Equations (2), (3), (4)) give the mathematical representation of the performance metrics.

(2) $$RMSE=\sqrt{\frac{1}{n}\sum_{i=1}^{n}{\left(Y_{i}-\dot{Y_{i}}\right)}^{2}}$$

(3) $$MAE=\frac{\sum_{i=1}^{n}\left|y_{i}-x_{i}\right|}{n}$$

(4) $$R^{2}=-\;\frac{\frac{1}{N}\sum_{I=1}^{N}{\left(y_{i}-{\hat{y}}_{i}\right)}^{2}}{\frac{1}{N}\sum_{I=1}^{N}{\left(y_{i}-{\bar{y}}_{i}\right)}^{2}}$$

### 4.2 Comparative prediction result

The regression equation derived is accurate within the bound of the temperature, volume fraction, and mixing ratio experimentally synthesized in this study. Equation (5) gives the correlation equation found in this work;

(5) $$\rho_{nf}=1186.42+69.73\varphi -153.39M_{R}-1.26T$$

Where $\rho_{nf}$, φ, $M_{R}$, and T represent the density of nanofluid, volume fraction, mixing ratio and temperature respectively. It is of worthy note that the mixing ratio used is the ratio of Al_2_O_3_ in the ternary nanofluid. The R^2^ and standard error estimated from the regression are 0.733 and 18.6 respectively.

**Fig. 10:**
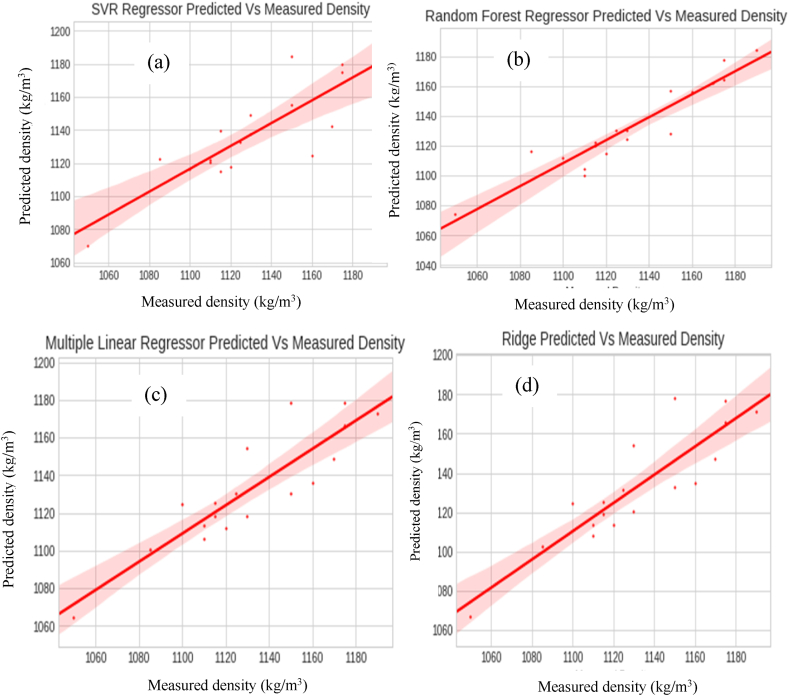
Testing results of Measured Vs Predicted Density of Ternary nanofluids (a) SVR (b) RF (c) MLR (d) RG algorithms.

**Fig. 11:**
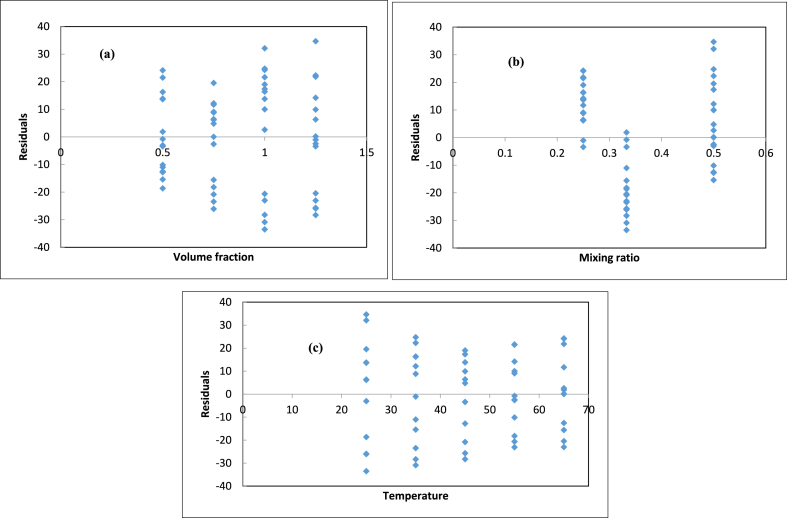
Residuals of the Multiple Linear regression for the experimental (a) Volume fraction (b)Mixing ratio (c) Temperature.

**Fig. 12:**
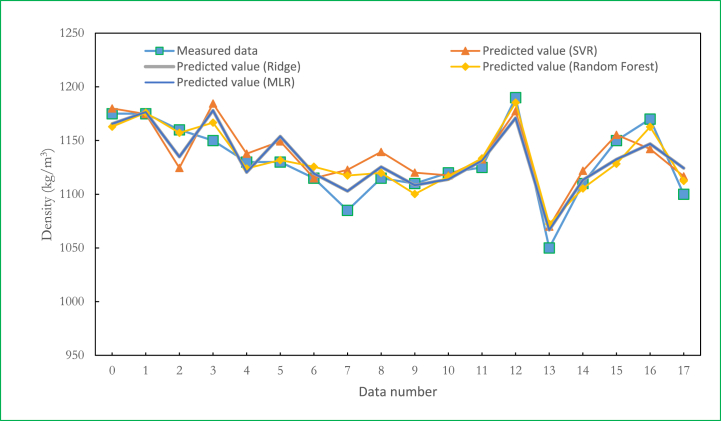
Comparison of all algorithms used in this study.

Table 3 and Fig. 10(a–d) show the results of the Multiple Linear Regression (MLR) and the machine learning algorithms' predictions, respectively. Table 3 shows that random forest regression models have the best prediction accuracy. The random forest regressor has an R^2^ of 0.928, while the SVR was observed to have the least accuracy, as it had the highest RMSE score of 19.541. Fig. 10 shows the measured and predicted data of the algorithms used in this work. Fig. 11(a–c) gives more specific residual values of the MLR predictions. Fig. 12 shows that the testing data for the random forest regressor were more closely aligned to the data line and the measured values (experimental data).

**Table 3:** Performance metric for the machine learning algorithms.

Model	RMSE	MAE	R^2^
Support Vector Regression (SVR)	19.541	15.496	0.826
RandomForest Regression (RF)	12.886	9.502	0.928
Multiple linear Regression (MLR)	16.062	13.678	0.864
Ridge (RG)	16.289	13.786	0.883

**Fig. 13:**
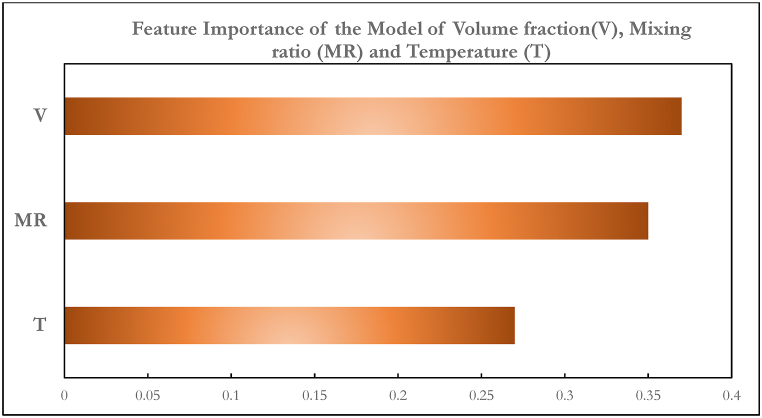
Feature importance of variables for density prediction.

The relative relevance of the factors used to estimate the nanofluid density is shown in Fig. 13. It is seen that the density of the nanofluid is most affected by the volume fraction. This is consistent with the results of density behaviour experiments performed on nanofluids. It's also worth noting that density was more affected by the mixing ratio than temperature. Even the little decrease in density with the increasing temperature of nanofluid has previously been fully elucidated in the literature [52]. This work underscores a critical insight from the prediction analysis: the volume fraction of nanoparticles involved in the synthesis of hybrid nanofluids significantly influences their density behaviour. For future ternary nanofluids to be useful, particularly in solar thermal applications, designers will need to optimize the mixing ratio of nanoparticles.

## 5 Conclusion

Hybrid nanofluids are becoming increasingly popular in vehicle fuel and coolant systems due to their enhanced thermal performance and faster heat transfer rates. Ternary-hybrid nanofluids, which are water-based and incorporate three distinct types of nanoparticles in a wide array of mixing ratios, present an intriguing yet largely theoretical concept. While there has been considerable research on the thermal conductivity, viscosity, and specific heat capacity of hybrid nanofluids, their density has been studied to a lesser extent.

This study presents a novel experimental analysis of the density of an Al_2_O_3_–ZnO–Fe_3_O_4_ ternary nanofluid. Some of the results from the experimental analysis include.

• At a temperature of 25 °C and a volume fraction of 1.25 %, the maximum density is determined to be 1165 kg/m^3^.
• Additionally, at 25 °C, 35 °C, 45 °C, 55 °C, and 65 °C, the density increases for the 1:1:1 mixing ratio at a volume fraction of 1.25 % is 14.4 %, 13.6 %, 13.13 %, 12.8 %, and 12.5 % respectively.
• Finally, the 1:1:1 exhibits the least density behaviour at increased volume fraction.
• The random forest algorithm gives the best prediction accuracy with an R^2^ of 0.928.

It is important for future studies to consider a more varied mixing ratio of nanoparticles in a ternary mixture, to give more clarity to the effect of combining different types of nanoparticles (also referred to as the hybridization effect [53]). Previous research has shown that the hybridization of nanoparticles can significantly alter their physical and chemical properties [34]. However, the mechanisms underlying these enhancements are not fully understood, and the optimal ratios of components in these hybrid systems remain largely unexplored. Therefore, investigating a broader range of mixing ratios could provide valuable insights into the synergistic effects, potentially leading to more effective nanomaterials for specific applications. Also, a range of particle sizes and shapes of the nanoparticles can be analyzed to understand their effect on the density of the ternary nanofluids. A key limitation in this study is the non-availability of a wider range of characterization techniques like the TEM analysis, however, the SEM analysis has been deemed sufficient to show the morphology of the nanocomposites.

## Ethical approval

Not Applicable.

## Consent to participate

Not Applicable.

## Consent to publish

The signed Consent to Publish permits the Publisher of the Author to publish the Work.

## Funding

This work was supported by the 10.13039/100009100Universiti Brunei Darussalam under grant number UBD/RSCH/1.3/FICBF(b)/2022/017.

## Data availability statement

Data used in this manuscript will be made available upon reasonable request.

## CRediT authorship contribution statement

**Humphrey Adun:** Writing – review & editing, Writing – original draft, Methodology, Conceptualization. **Muhammad Abid:** Supervision. **Doga Kavaz:** Formal analysis. **Yihua Hu:** Investigation, Data curation. **Juliana Hj Zaini:** Writing – review & editing.

## Declaration of competing interest

The authors declare that they have no known competing financial interests or personal relationships that could have appeared to influence the work reported in this paper.

## Nomenclature

DLS: Dynamic Light Scattering
EDX: Energy Dispersive X-Ray Analysis
EG: Ethylene glycol
FTIR: Fourier transform infrared
g: gram
HTF: Heat transfer fluid
MAE: Mean absolute Error
ml: Millilitre
MLR: Multiple Linear Regression
$M_{R}$: Mixing ratio
RG: Ridge
RMSE: Root Mean Squared Error
SEM: Scanning electron microscopy
SVR: Support Vector Regression
T: Temperature
TEM: Transmission electron microscopy
PCS: Photon Correlation Spectroscopy
RF: Random Forest
R2: coefficient of determination
φ: Volume fraction
ρ: Density
$\rho_{\text{nf}}$: Density of nanofluid
XRD: X-ray diffraction
